# Analyzing On-Farm Spatiotemporal Distribution of *Halyomorpha halys* (Stål) (Hemiptera: Pentatomidae) Populations from a Precision Agriculture Perspective

**DOI:** 10.3390/plants12122282

**Published:** 2023-06-12

**Authors:** Vasileios Liakos, Ioannis E. Navrozidis, Eleni I. Koutsogeorgiou, Nikoloz E. Gogolashvili, Eugenia Samourgkanidou, Ioannis Faraslis, Ioannis Gravalos, Thomas Thomidis, Stefanos S. Andreadis

**Affiliations:** 1 Laboratory of Precision Agriculture, Department of Agrotechnology, University of Thessaly, Gaiopolis, 41110 Larissa, Greece; vliakos@uth.gr (V.L.); iogravalos@uth.gr (I.G.); 2 Laboratory of Remote Sensing, Spectroscopy and GIS, School of Agriculture, Faculty of Agriculture, Forestry and Natural Environment, Aristotle University of Thessaloniki, 54124 Thessaloniki, Greece; navrozidisie@gmail.com; 3 Laboratory of Applied Zoology and Parasitology, School of Agriculture, Faculty of Agriculture, Forestry and Natural Environment, Aristotle University of Thessaloniki, 54124 Thessaloniki, Greece; eikoutso@agro.auth.gr; 4 Institute of Plant Breeding and Genetic Resources, Hellenic Agricultural Organization-Dimitra, P.O. Box 60458, 57001 Thermi, Greece; 5 Department of Agriculture, International Hellenic University, 57400 Sindos, Greece; gogolanick@gmail.com; 6 Department of Biology, Aristotle University of Thessaloniki, 54124 Thessaloniki, Greece; eugenia-s.27@hotmail.com; 7 Department of Environment, University of Thessaly, Gaiopolis, 41110 Larissa, Greece; faraslis@uth.gr; 8 Department of Nutritional Sciences & Dietetics, International Hellenic University, 57400 Sindos, Greece; thomidis@ihu.gr

**Keywords:** brown marmorated stink bug, pheromone traps, captures, terrain, canopy, indices, satellites

## Abstract

The brown marmorated stink bug, *Halyomorpha halys* Stål (Hemiptera: Pentatomidae), is native to Japan, China, Taiwan, and Korea. Its dispersion from Asia to the United States of America and Europe caused serious damage to fruit, vegetables, and high-value crops. In Greece, damages are reported in kiwi orchards in the regions of Pieria and Imathia, which are the main production areas of kiwifruit. Greek kiwifruit production is expected to increase twofold within the next years. The aim of this research is to study the terrain and canopy properties that may have an impact on the development of *H. halys* populations. Thus, five kiwi orchards in total were selected in the regions of Pieria and Imathia. Τen traps were installed from early June to late October within each selected kiwi orchard–two types of traps at every side of the orchards and the center. The installed traps were examined weekly and the number of the captured *H. halys* was recorded. During the same days, sentinel satellite images were analyzed to calculate the vegetation index, NDVI (Normalised Difference Vegetation Index), and NDWI (Normalised Difference Water Index). The results showed population variability within the kiwi orchards since the population of *H. halys* was higher in areas with high NDVI and NDWI values. Additionally, our research revealed that *H. halys* prefers to develop populations at higher altitudes at both regional and field scales. The results of this research can be used to reduce damages by *H. halys* in kiwi orchards using different rates of pesticides depending on the prediction of the population size. There are multiple benefits of the proposed practice, such as a reduction in the production cost of kiwifruits, an increase in farmers’ profit, and environmental protection.

## 1. Introduction

The brown marmorated stink bug (BMSB), *Halyomorpha halys* Stål (Hemiptera: Pentatomidae), is a highly polyphagous agricultural pest that was accidentally introduced in the United States and Europe from East Asia [1,2,3]. This pest rapidly develops high populations on a variety of host plants within and near orchards and crop fields causing significant economic losses to farmers [4,5]. The introduction of *H. halys* resulted in severe damage to apple, peach, and pear orchards [1,6]. In Europe, a few years since its first discovery in Liechtenstein in 2004 [7], *H. halys* became the main key pest in various fruit orchards [8,9,10,11,12], vegetable crops [13], kiwifruit [14] and hazelnuts [15]. In addition, *H. halys* is considered a severe nuisance pest for homeowners due to the fact that overwintering adults disperse to and settle within residential buildings and other human-made structures in large numbers during autumn [3,16].

*Halyomorpha halys* resides in different crops depending on the season. For example, adults can be found in natural areas [17] and fabricated structures in winter [16] and in crop fields in spring and summer [18]. Adults are capable of both short- and long-range dispersal [19,20,21], depending on the presence of host plants, as well as their availability [22]. However, a high level of crop damage occurs at the edges of fields that are near unmanaged forests or uncultivated fields [23]. The abundance of wild and cultivated host plants contributes to the development of large populations of *H. halys* [4,24]. The spatial distribution of *H. halys* may change over the course of a season. At the beginning of each season, *H. halys* leave their overwintering sites to find food from available early-season host plants. After that, they establish their population on the host plants and then they disperse from one habitat to another. However, few articles describe its overwintering ecology in its native range [25] and report in isolated and general observations that adults can overwinter in ground litter and inside tree holes [26], under tree bark [27,28], and in dry, high-elevation mountains [17,29]. In Northern Greece in particular, *H. halys* has been reported to experience facultative reproductive diapause during unfavourable conditions [30].

*Halyomorpha halys* is considered a perimeter-originated threat, which is why many researchers focus on developing new strategies for insecticide application at crop field borders rather than across fields [31,32,33,34,35]. Most studies use data from pheromone traps placed only at crop field boundaries rather than throughout the field to monitor *H. halys* populations [36,37,38].

Timely and accurate detection of kiwi canopy characteristics may be necessary to detect canopy damage caused by *H. halys* and subsequent economic losses. Early detection of *H. halys* infestations in kiwifruit orchards could be improved by multispectral remote sensing. Multispectral remote sensing has the potential to provide information on pest status in fields and has been used to detect pest-induced crop damage in sorghum [39], cotton, and wheat [40,41,42,43,44]. The Normalised Difference Vegetation Index (NDVI) is a critical parameter for identifying the condition of vegetation and detecting plant damage. The NDVI defines values from −1 to 1, with negative values formed mainly by clouds, water, and snow, and values close to zero formed by bare soil. Moderate NDVI values represent areas devoid of vegetation, while large NDVI values (near 1) indicate healthy vegetation. A range of NDVI information derived from remote sensing imagery has resulted in NDVI time series from remote sensing data becoming an important source of information for crop monitoring [45,46]. However, there is limited research linking NDVI to insect presence. Willers et al. [47] established a spatial relationship between the hemipteran insect *Lygus lineolaris* and areas of high cotton growth (NDVI). The Normalised Difference Water Index (NDWI) is another critical parameter that has a strong relationship with plant leaf water content. NDWI can take values between −1 and 1 and responds more quickly than NDVI to changes in water availability. When leaf water content decreases, NDWI values also decrease and reverse. This index has been used to investigate the remote sensing potential for mapping and monitoring vegetation water content in corn and soybean canopies [48], to characterise land cover and vegetation type [49,50], and last but not least, to monitor water stress in semiarid areas [51]. As with NDVI, there are few studies on the use of NDWI and pest control. McFeeters et al. [52] used NDWI in conjunction with geographic information systems to detect swimming pools to reduce mosquito populations. Jokar et al. [53] studied the distribution of cotton bollworm, *Helicoverpa armigera* (Lepidoptera: Noctuidae), and created inundation maps using NDWI data from the Sentinel 1 satellite. Finally, Prabhakar et al. [54] successfully detected invasive fall armyworms by extracting NDWI data from Sentinel 2A satellite imagery.

Management of *H. halys* in commercial fields relies mainly on the use of broad-spectrum insecticides. However, the pesticides that are effective against *H. halys* are often toxic to pollinators and natural enemies. For this reason, farmers and other stakeholders are seeking new strategies to reduce the use of pesticides to control *H. halys* populations. According to Blaauw et al. [31], several pest management programmes were never implemented due to overuse of chemicals. A method for controlling *H. halys* populations with evident results is to spray high amounts of chemicals in areas with high *H. halys* populations and reduce the amount of chemicals in areas with low density of *H. halys* populations [55]. This strategy is consistent with precision agriculture, which recommends the use of agrochemicals at the right dosage and in the right place, depending on crop needs. It is worth noting that the discovery of aggregation pheromones of *H. halys*, which are a combination of the stereoisomers (3S,6S,7R,10S)-10,11-epoxy-1-bisabolen-3-ol and (3R,6S,7R,10S)-10,11-epoxy-1-bisabolen-3-ol (PHER), and a synergist, methyl (2E,4E,6Z)-2,4,6-decatrienoate (MDT), the aggregation pheromone of *Plautia stali* (Scott) (Hemiptera: Pentatomidae), opened up the possibility of developing baits that attract this pest to the vicinity of the baited position. The lures are combined with sticky, rocket, or pyramid traps to help farmers determine the presence and density of *H. halys* populations. Commercially available pheromone traps specialised for *H. halys* capture males, females, and nymphs [36,56,57,58,59]. Traps tagged with this commercial double bait have been used to capture *H. halys* to study its spatial distribution in different agroecosystems [22,23]. However, it would be more efficient for farmers to use a decision support tool that predicts the areas where *H. halys* will form populations.

This study presents *H. halys* population data collected with pheromone traps during two growing seasons in Greece, as well as the corresponding NDVI and NDWI data obtained from Sentinel-2 imagery. These data were spatially and temporally analyzed together with the characteristics of the region and the terrain. The goal of this research is to find plant and terrain characteristics that are useful in predicting *H. halys* populations spatially and temporally. The results will be used by farmers to apply insecticides at variable rates rather than the usual uniform rates in order to reduce the amount of insecticides applied, increase their profits, and protect the environment and other beneficial insects. The European Union subsidizes the use of variable rate applications of insecticides through precision agriculture funding programs. The objectives of this study are to (a) understand the preferences and needs of *H. halys* in three different seasons, (b) investigate whether the spatial and temporal patterns of *H. halys* population dynamics are controlled by environmental factors that can be remotely observed using NDVI and NDWI, and (c) demonstrate whether the variability of terrain and elevation characteristics within a field have an effect on *H. halys* population trends.

## 2. Results

### 2.1. Captures of Adults and Nymphs

**Figure 1:**
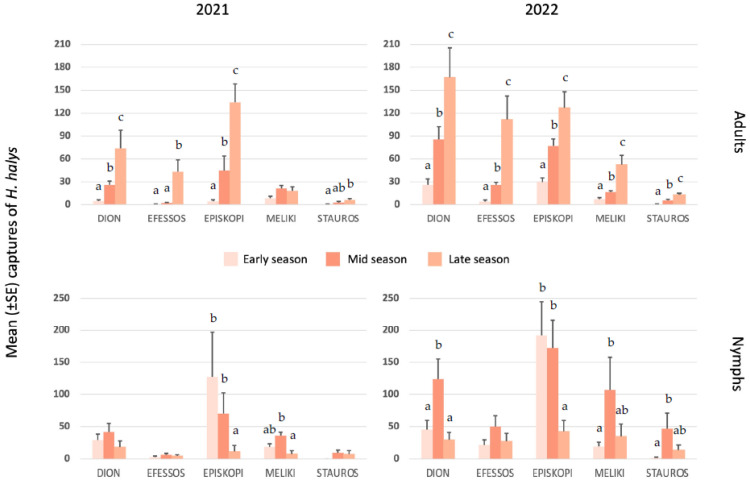
Weekly captures of *H. halys* nymphs and adults across trap types and aggregated for 2021 and 2022 at each experimental site (Dion, Nea Efessos, Episkopi, Meliki, and Stauros). Data divided into three different groups/seasons—in the early-, mid-, or late-season to understand *H. halys* temporal behaviour and preferences. Bars with different lowercase letters (within each experimental site) are significantly different from each other (Tukey’s honestly significant difference, *p* < 0.05).

Significant differences in the number of adult *H. halys* captured in 2021 and 2022 in the three seasons at each site were observed (Figure 1). More specifically, in 2021 there were significant differences between adults captured in the late season and those captured in the early and mid-season (Dion, Nea Efessos, Episkopi). In addition, significant differences were found between adults caught in the late and early seasons (Stauros). No significant differences were found in adults caught in Meliki during 2021. As in 2021, significant differences were found in adults captured in the late season and those captured in the early and mid-season in 2022 (Dion, Nea Efessos, Episkopi, Meliki, and Stauros). On the other hand, significant differences were found only in nymphs captured in the late season and those captured in the early/mid-season (Episkopi) as well as between those captured in mid-season and late season (Meliki) of 2021. Significant differences were also found in the number of nymphs caught in 2022 between the mid-season and the other two seasons (Dion), between the early season and the mid-season (Stauros), and between the late season and the other two seasons (Meliki and Episkopi). Figure 1 shows the distribution of *H. halys* captures in three seasons–early, mid, and late for both years. The number of adults and nymphs caught is low at the beginning of the growing season. The population of adults was higher in the late season than in the other two seasons (except in Meliki in 2021). For nymphs, the highest number was recorded in mid-season compared to the other two seasons (except for Episkopi in both years). On the other hand, the number of nymphs was lowest in the late season in both years. The highest number of adults was recorded in Episkopi (late season 2021) and Dion (late season 2022), and the lowest in Stauros in both years. The highest number of nymphs was counted in Episkopi in the early season in both years and the lowest in Nea Efessos in 2021 and Stauros in 2022.

Figure 1 shows that the number of adults and nymphs caught in the late season was higher than in previous years, but catches were still low in some areas (2021 in Stavros and Nea Efessos and 2022 in Stavros). The presence of adults was linearly correlated with the presence of nymphs in 2021 (r = 0.96, *p* < 0.01). In 2022, there was no correlation between the number of adults and nymphs captured (r = −3.61, *p* > 0.05).

### 2.2. H. halys Populations, Terrain Properties, and Indices

**Figure 2:**
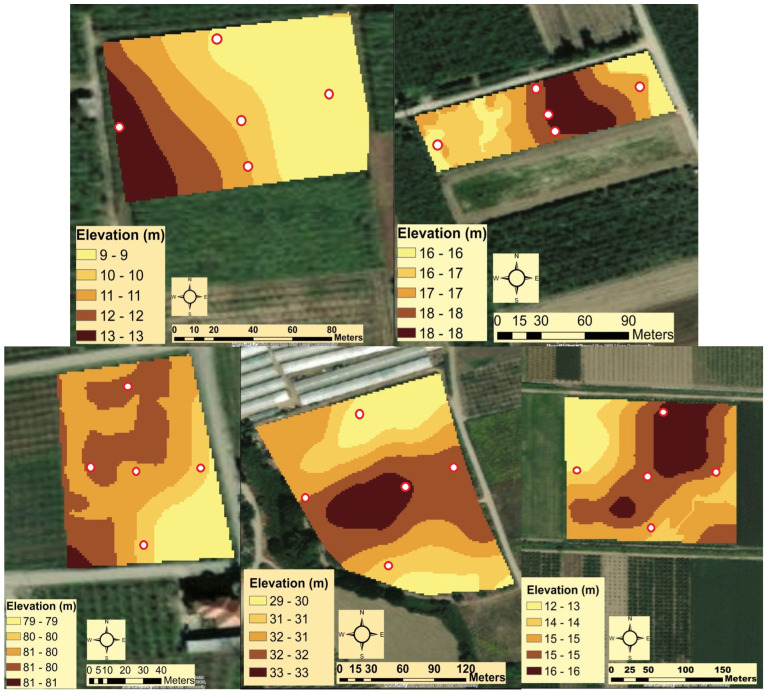
From left to right: Elevation maps from the sites Dion, Nea Efessos, Episkopi, Meliki, and Stauros, respectively. The darker the colour on the maps the higher the elevation. White colour points with red outlines present the trap locations.

Figure 2 shows the variability of the terrain within the experimental sites. The sites in Episkopi and Nea Efessos have small differences in elevation compared to the other three sites. In addition to the low elevation differences, the spatial variability of the terrain is very high. The use of the Geostatistical Analyst tool from ArcGIS (ESRI, Redlands, CA, USA) showed that the percentage coefficient of variation percent (CV %) of the elevation data within each field is about 60% and the data follow a normal distribution (0, 50, 7).

**Figure 3:**
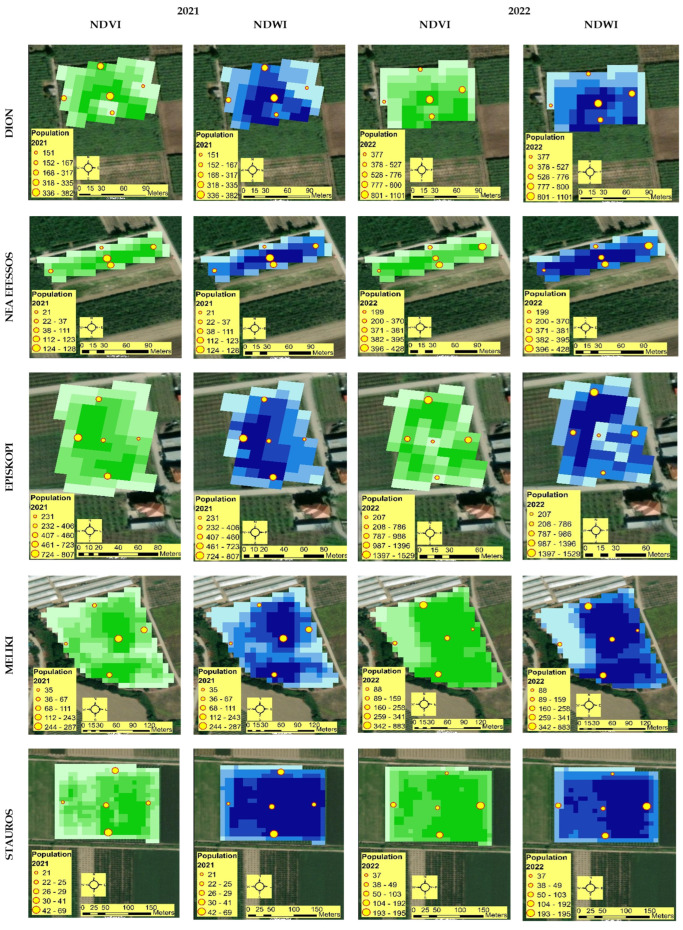
NDVI, NDWI, and *H. halys* spatial variability in both years of the research. Green maps present the NDVI variability. The darker the green the higher the NDVI values and the opposite. Blue maps present the NDWI variability. The darker the blue the higher the moisture at the canopy and the opposite. The yellow circles with red outlines show the exact location of the installed traps. The size of the yellow circles represents the size of the *H. halys* population. The bigger the circle the bigger the population.

Mapping of NDVI and NDWI variability contributed to the understanding of variability in each field (Figure 3). The classification method used to map the variability of the indices is the quantile method. This method assigns the same number of data values to each class. Visual comparison of the size of *H. halys* populations at different sites relative to the values of vegetation indices at those specific sites demonstrates that *H. halys* tends to develop populations at sites where the plant canopy is healthy (high NDVI values) and moisture is present in the canopy (high NDWI values).

Statistical analysis of H. halys populations, height characteristics, NDVI, and NDWI showed that there was a high correlation between them in both survey years (Table 1). This means that H. halys tends to develop populations in higher elevation areas where the plant canopy is healthy (high NDVI values) and moisture is present in the canopy (high NDWI values).

The occurrence of nymphs caught in the traps in each growing season depends on the altitude of the orchard. The higher the elevation, the earlier the nymphs appear in the orchards. According to Table 2, nymphs in kiwi orchards are caught in early June in the higher elevation areas and from mid-June in the lower elevation areas.

## 3. Discussion

The two-year use of pheromone-baited traps to monitor and capture *H. halys* individuals revealed spatiotemporal changes within kiwi orchards during growing seasons. The collected data were divided into three equal sampling periods based on annual phase changes in *H. halys* populations. Few adults were captured at the beginning of each season, but as the season progressed, adult captures tended to increase throughout September and October. This is evident in Figure 1, which shows significant differences in adults caught in the late season compared to the early and late seasons. In contrast to other studies [35,36,37], nymphs were present in every season, not just in the mid and late seasons. Adults showed a different distribution in the mid and late-season, but their distribution in the mid-season was almost the same in each field in both years. Hahn et al. [60] showed similar results for adult and nymph captures of *H. halys*. High populations of adults and nymphs of *H. halys* were recorded in the mid and late seasons, as fruit is more susceptible to damage by stink bugs during this time [6]. In the current study, *H. halys* individuals captured with traps were fewer in 2021 than in 2022, possibly due to the fact that there was a spring frost early in the season in 2021, which reduced host plant fruit set [61] and consequently affected the development of *H. halys* populations throughout the growing season [62]. The presence of adult *H. halys* populations and nymphs later in the season and especially after harvest suggests that adults may overwinter in or near orchards [30,63,64]. These results are very important considering that the study sites were geographically distributed among locations with different climates and topography.

Regional elevation differences and altitudinal variation within orchards provided evidence that terrain influences *H. halys* populations. Regional elevation plays an important role in the time period during which nymphs are active. The higher the elevation, the earlier the emergence. This suggests that *H. halys* is active and seeks overwintering sites earlier at higher elevations than at lower elevations. Similar results were obtained by Cullum et al. [17]. Terrain variability affects the establishment of *H. halys* populations, as *H. halys* prefers higher elevations even within a small orchard. Cullum et al. [17] reported similar results for *H. halys* mainly at the regional level rather than at the orchard level.

There are few studies on *H. halys* in kiwifruit orchards using satellite imagery to extract information on vegetation characteristics such as NDVI and NDWI. Zhu et al. [65] developed a model based on NDVI and other data to predict the spatial distribution of *H. halys* at a large scale. Reisig et al. [66] used NDVI data to estimate the damage already caused by stink bugs to cotton plants. The current work showed that elevation is significantly correlated with NDVI and NDWI. NDVI provides information on kiwi canopy vigour and density. Higher NDVI values mean that the canopy is greener and has a high density of leaves, creating shade under the plants. This is consistent with the results of other studies that concluded that *H. halys* prefers dark areas over light areas [17,21,67]. Moreover, in the current study, more *H. halys* were trapped in the areas where NDWI was high, i.e., where canopy moisture was high. This is in contrast to Cullum et al. [17], who reported that *H. halys* preferred dry rather than moist areas.

From the above, it is clear that NDVI, NDWI, and elevation play a very important role in the establishment of *H. halys* populations. Knowing the factors that affect *H. halys* populations, as well as the timing and location of *H. halys* presence, can help farmers manage their orchards efficiently by applying agrochemicals or natural enemies at the right time and place according to precision agriculture practices, rather than uniformly as they usually do. These new precision agriculture practices typically reduce production costs and also protect the environment. The next step in this study is to develop a smart app for smartphones that will allow users to access elevation, NDVI, and NDWI data. This app will be programmed to dynamically predict locations at high risk for *H. halys* occurrence in fields and alert users via email or phone notification to take further action at specific locations.

## 4. Materials and Methods

### 4.1. Field Sites

In 2021 and 2022, a field survey was conducted on five kiwifruit orchards located at Dion, Nea Efessos, Episkopi, Meliki, and Stauros in the prefectures of Imathia and Pieria, in Greece. Table 3 shows the field characteristics of each kiwifruit orchard. All kiwifruit orchards were uniformly irrigated and fertilised. Farmers treated kiwifruit orchards without insecticides against *H. halys*, which provided an opportunity to detect seasonal changes in *H. halys* at several locations. The kiwi variety ‘‘Haywarth’’ was cultivated in all kiwifruit orchards.

The crop type from fields near the experimental orchards was recorded in both years to examine the effects of these crops on *H. halys* populations.

### 4.2. Traps

The trap types used for monitoring *H. halys* included the small hanging rocket trap (Rescue^®^ stink bug trap; Serbios S.r.l., Badia Polesine, Italy) as well as the sticky panel trap (Trécé Inc.), both installed in the tree canopy, baited with the available standard lures commercially produced by Rescue^®^ or Trécé Inc, respectively. In total, ten (10) traps (five of each type) were deployed at each orchard. One trap of each type was installed at each side and the centre of each field. The traps were inspected weekly and the number of the captured adult and nymphs of *H. halys* was recorded from early June to late October in both years.

### 4.3. Plant Canopy Characteristics

Sentinel satellite images were acquired every five days and analysed to calculate the Normalised Difference Vegetation Index (NDVI) as well as the Normalised Difference Water Index (NDWI). NDVI [66] is calculated using Equation (1) and NDWI [52] is calculated using Equation (2). NDVI values provide information about the vigour and the density of a canopy. NDVI values close to 1 indicate that the canopy is vigorous, while NDVI values close to or below 0.2 indicate deficiencies in the canopy. On the other hand, the NDWI value indicates the moisture content of the canopy. The higher the NDWI value, the higher the moisture content. After acquiring satellite images from June to October of each year and at the end of each growing season, NDVI and NDWI maps were created by averaging the pixel values of NDVI and NDWI values from all satellite images. The spatial resolution of the NDVI and NDWI maps was 10 m, as this was the spatial resolution of the Sentinel satellite imagery. Spatial analysis and mapping of average NDVI and NDWI values were performed using ArcGIS (ESRI, Redlands, CA, USA). The final NDVI and NDWI maps for each season were generated using the Map Algebra (raster calculation) tool of ArcGIS.

(1) $$NDVI=\frac{NIR-RED}{NIR+RED}$$

(2) $$NDWI=\frac{GREEN-NIR}{GREEN+NIR}$$

where: NIR is the reflectance at the near-infrared band.

RED is the reflectance at the red band.

GREEN is the reflectance at the green band.

### 4.4. Terrain Properties

Elevation data of each kiwi orchard were recorded using a Real-Time Kinematic Global Navigation Satellite System (Neo-M8P RTK GNSS: Ublox, Thalwil, Switzerland). This system provides very precise information for location identification on the earth, but it also provides very precise data about the elevation of users. The spatial accuracy of the system was about 5 cm on the days of data collection. ArcGIS was used to create the Digital Elevation Model for each site. Data analysis such as Tukey post hoc analysis and Pearson’s correlation (r) of the captured adult and nymph *H. halys* data was performed using IBM SPSS Statistics (Version 29). Statistical analysis focused on the correlation between variables (terrain, NDVI, and NDWI) and population densities of *H. halys* using the linear regression tool of the software. Each growing season was split up into three sampling periods with roughly equal trapping intervals to improve statistical analysis and understand the temporal preferences of *H. halys* [9,32,33,36,37,38,68]. Early season refers to the period from early June to late July, mid-season from late July to early September, and late season from early September to mid-October. The calculation of the mean number of captures and the standard error for each season was performed using IBM SPSS Statistics.

## Figures and Tables

**Table 1:** Correlation between population densities, topography, and indices extracted from satellite images.

	2021				2022			
	Density	Elevation	NDVI	NDWI	Density	Elevation	NDVI	NDWI
Density	1				1			
Elevation	0.84 **	1			0.88 **	1		
NDVI	0.78 **	0.62 **	1		0.86 **	0.77 **	1	
NDWI	0.81 **	0.68 **	0.69 **	1	0.85 **	0.75 **	0.73 **	1

** Correlation is significant at the 0.01 level.

**Table 2:** Dates of first *H. halys* nymphs captures in relation to site elevation (m).

Region	Date 2021	Date 2022	Elevation
Dion	16 June	15 June	9–13
Nea Efessos	23 June	15 June	16–18
Episkopi	10 June	01 June	78–81
Meliki	10 June	8 June	29–33
Stauros	27 June	29 June	12–16

**Table 3:** Coordinates in decimal degrees, elevation above sea level in meters, orchard size in hectares, and soil type for each kiwi orchard.

Prefecture	Locations	Longitude	Latitude	Elevation (m)	Size (ha)	Soil Type
Pieria	Dion	22.491933	40.170259	13	0.69	Silt
	Nea Efessos	22.489333	40.221147	18	0.56	Silt
Imathia	Episkopi	22.127619	40.689933	80	0.70	Silt
	Meliki	22.404548	40.519695	34	2.36	Silt
	Stauros	22.310885	40.565675	15	3.32	Silt

## Data Availability

The data presented in this study are available on request from the corresponding author.
